# Oxygen‐Rich Carbon Nitrides from an Eutectic Template Strategy Stabilize Ni, Fe Nanosites for Electrocatalytic Oxygen Evolution

**DOI:** 10.1002/advs.202300526

**Published:** 2023-05-28

**Authors:** Chun Li, Enrico Lepre, Min Bi, Markus Antonietti, Junwu Zhu, Yongsheng Fu, Nieves López‐Salas

**Affiliations:** ^1^ Key Laboratory for Soft Chemistry and Functional Materials of Ministry of Education Nanjing University of Science and Technology Nanjing 210094 China; ^2^ Colloid Chemistry Department Max Planck Institute of Colloids and Interfaces Am Mühlenberg 1 14476 Potsdam Germany

**Keywords:** carbon nitrides, eutectic mixtures, hard templating, oxygen evolution reaction

## Abstract

Functionalized porous carbons are central to various important applications such as energy storage and conversion. Here, a simple synthetic route to prepare oxygen‐rich carbon nitrides (CNOs) decorated with stable Ni and Fe‐nanosites is demonstrated. The CNOs are prepared via a salt templating method using ribose and adenine as precursors and CaCl_2_·2H_2_O as a template. The formation of supramolecular eutectic complexes between CaCl_2_·2H_2_O and ribose at relatively low temperatures facilitates the formation of a homogeneous starting mixture, promotes the condensation of ribose through the dehydrating effect of CaCl_2_·2H_2_O to covalent frameworks, and finally generates homogeneous CNOs. As a specific of the recipe, the condensation of the precursors at higher temperatures and the removal of water promotes the recrystallization of CaCl_2_ (*T* < *T_m_* = 772 °C), which then acts as a hard porogen. Due to salt catalysis, CNOs with oxygen and nitrogen contents as high as 12 and 20 wt%, respectively, can be obtained, while heteroatom content stayed about unchanged even at higher temperatures of synthesis, pointing to the extraordinarily high stability of the materials. After decorating Ni and Fe‐nanosites onto the CNOs, the materials exhibit high activity and stability for electrochemical oxygen evolution reaction with an overpotential of 351 mV.

## Graphical abstract

A new eutectic‐mediated salt templating method enables the synthesis of oxygen‐rich carbon nitrides (CNOs) with high oxygen and nitrogen contents (12 and 20 wt%, respectively) while maintaining exceptional material stability up to 800 °C. The CNOs decorated with Ni and Fe‐nanosites display impressive activity and stability for electrochemical oxygen evolution (351 mV overpotential).

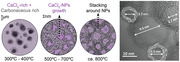

## 1 Introduction

Porous carbon materials are essential in many important applications because of their wide availability and superior physicochemical properties,^[^ ^1^ ^]^ such as electric and thermal conductivities, chemical stabilities, low densities, as well as their high specific surface area and adjustable pore size. For example, they have been widely used as electrodes for fuel cells,^[^ ^2^ ^]^ supercapacitors,^[^ ^3^, ^4^ ^]^ and batteries,^[^ ^5^ ^]^ as well as effective supports for separation,^[^ ^6^ ^]^ sorption,^[^ ^7^ ^]^ gas storage,^[^ ^8^ ^]^ and catalysis.^[^ ^9^ ^]^ In particular, porous carbon materials with targeted functionality are one of the most competitive materials compared to other porous materials, such as metal‐organic frameworks, porous silica, porous polymers, and zeolites, because of their abundant, inexpensive raw materials and thus great economic attraction.^[^ ^10^, ^11^ ^]^ An efficient way of functionalization is attaching heteroatoms (e.g., doped with nitrogen, oxygen, sulfur) directly during their synthesis.^[^ ^12^ ^]^ To fulfill multiple, combined functions, for example, high activity and stability in electrocatalysis, heteroatom‐doped porous carbons often cooperate with metallic nanoparticles/nanosites. They use metal‐carbon interaction resulting from the long electron pairs of the heteroatoms with the empty orbitals of metals to stabilize metallic sites in a ligating fashion^[^ ^13^, ^14^, ^15^, ^16^ ^]^ and prevent them from aggregation.^[^ ^17^, ^18^, ^19^ ^]^ This allows the dispersion of a larger amount of stable metallic active sites, providing highly active and long‐lifetime catalysts.^[^ ^20^, ^21^ ^]^

The heteroatom content and pore structure of heteroatom doped‐porous carbons can be altered by changing precursors and pyrolysis temperatures,^[^ ^22^, ^23^, ^24^ ^]^ and by using different physical and chemical methods, such as templating methods,^[^ ^25^ ^]^ salt melt synthesis, 3D printing,^[^ ^26^ ^]^ and freeze‐drying‐assisted synthesis.^[^ ^27^ ^]^ For example, Zhang et al. reported the in situ polymerization and carbonization of quinone‐amine polymer precursor on the surface of a nanosized MgO template to obtain hierarchical porous carbon.^[^ ^28^ ^]^ Moreover, the salt templating approach is simple and efficient, based on a salt acting as solvent and template. Thus, the crosslinking of the precursor and carbonization can proceed in one step. Some metal chlorides, like zinc chloride, were able to form eutectic mixtures with simple organic alcohols or amides, leading to a highly homogeneous reaction environment.^[^ ^29^ ^]^ Xue et al. fabricated N/O doped hollow carbon nanorods (HCNs) based on a new deep‐eutectic‐solvent (DES) formed via the hydrogen bond between urea, 2,5‐dihydroxy‐1,4‐benzoquinone, and ZnCl_2_. The new DES acts as a C/N/O precursor and auto‐template, while ZnCl_2_ can promote the condensation process due to its Lewis‐acidic dehydration effect.^[^ ^30^ ^]^ Other alkali metal chlorides such as LiCl, NaCl, and KCl^[^ ^31^ ^]^ were also well studied in salt melts and hard templating systems. There are only a few reports introducing alkaline earth metal chlorides such as CaCl_2_ and MgCl_2_ as salt templates.^[^ ^32^, ^34^ ^]^ Huang et al. investigated the multiple roles of CaCl_2_, where CaCl_2_ was finally encapsulated in nitrogen‐doped carbons, and a mesopores structure with high surface areas was obtained after aqueous removal of CaCl_2_. Pampel et al. described MgCl_2_·6H_2_O as both soft and hard templates in the construction of tubular nitrogen‐doped porous carbons with adenine as a precursor.^[^ ^35^ ^]^ The soft template comes from molten hydrate (eg. its hexahydrate, melting point 96 °C), while a hard template forms later due to the dehydration of the molten hydrate that then crystallizes. The interaction between MgCl_2_ hydrates and the precursors, however, was hardly discussed, and the mentioned literature neither discussed the mechanism of CaCl_2_ hydrates as templates.

Herein, we use CaCl_2_·2H_2_O as a salt template and ribose and adenine as precursors for the synthesis of oxygen‐rich carbon nitrides (CNOs) and found that CaCl_2_·2H_2_O not only acted as a template but also formed a supramolecular structure with ribose via hydrogen bonding at lower temperatures, providing the formation of a homogeneous eutectic mixture where condensation and carbonization processes occur. The solidification of CaCl_2_ (*T* < *T_m_*) at higher temperatures occurs during the condensation of the precursors, generating organized nanocrystals as hard templates. The CNOs nitrogen and oxygen contents were up to 20 and 12 wt%, respectively, even when the pyrolysis temperature was as high as 800 °C. A large number of mesopores with unique graphitic conjugated rings were observed in the CNOs sheets. The structure and elemental composition of these CNOs are extremely stable, making them candidates to perform substrates for metal deposition. The CNOs exhibit a higher loading capacity of nickel atoms than iron atoms, and CNOs with Ni exhibited better catalytic performance in oxygen evolution reaction (OER) than CNOs with Fe atoms. Interestingly, the addition of Fe atoms into Ni‐based CNOs displayed an enhancement in electrocatalytic activity, which could contribute to the synergistic effect between nickel and iron, located at different sites but connected via strands of carbon covalency. Due to the described features, the catalyst exhibited excellent durability.

## 2 Results and Discussion

### 2.1 Synthesis Mechanism of CNO

**Scheme 1:**
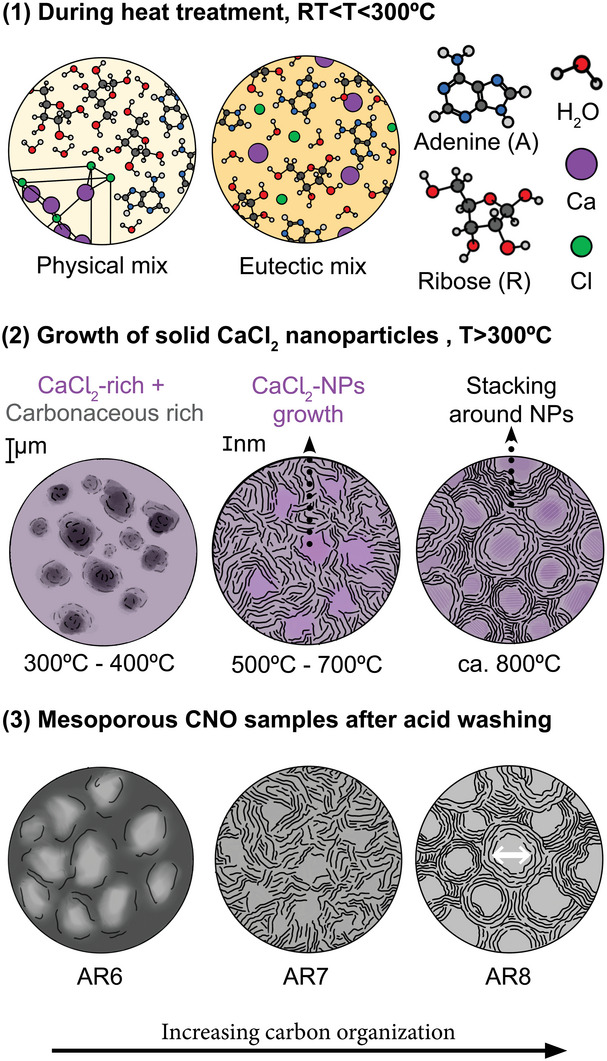
Schematic diagram of the formation process of CNOs.

The CNOs were synthesized as described above. The final oxygen and nitrogen contents of these CNOs are as high as 12 and 20 wt% and remain stable in a temperature range from 600 to 800 °C. Carbon yields are unusually high, speaking for tightly bound precursors. We found that the hydrogen bond between ribose and CaCl_2_·2H_2_O allows the formation of a supramolecular, liquid, eutectic structure. Moreover, the strong dehydrating effect of CaCl_2_·2H_2_O quickly activated and polycondensed the precursors and fostered the formation of CNOs with homogeneous morphology and structure. As shown in **Scheme** **1**, CaCl_2_·2H_2_O and ribose form a supramolecular structure that integrates into pre‐defined intermediates adenine, thus generating partially‐ordered, nanostructured carbons with stable elemental composition and properties.

**Figure 1:**
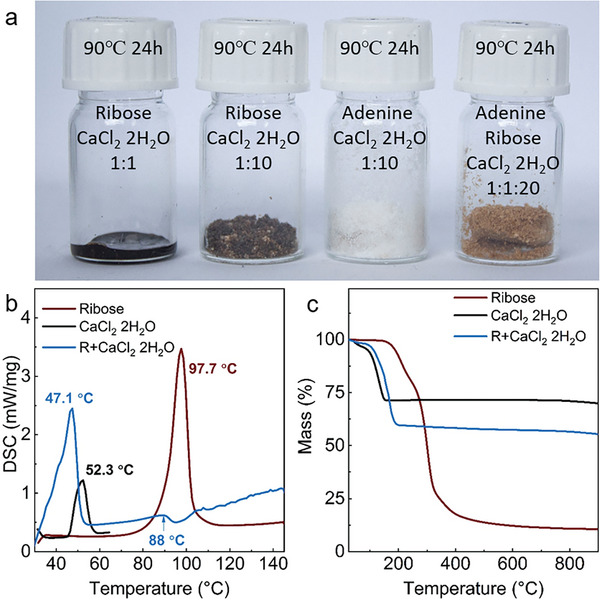
a) Digital pictures of mixed ribose, adenine, and CaCl_2_·2H_2_O (1:1 in mass) in a 90 °C oven for 24 h, b) DSC curves for melting point determination, and c) TGA curves of precursors and the mixture with CaCl_2_·2H_2_O.

As shown in **Figure** **1**a and Figure S1a, Supporting Information, the mixture of ribose and CaCl_2_·2H_2_O (the first bottle) turned into an organized, opaque liquid at 90 °C after 5 and 24 h, suggesting the formation of supramolecular structure since the vial was capped and thus the dehydration of CaCl_2_·2H_2_O could not take place. The supramolecular structure is formed via the hydrogen bond between ribose and CaCl_2_·2H_2_O, which can be proven by the DSC curves in Figure 1b. The DSC curve of ribose and CaCl_2_·2H_2_O mixture shows three peaks. The first one is related to the loss of adsorbed water because there is a mass loss in Thermogravimetric analysis (TGA) curves (Figure 1c). The second one is related to the phase change of the mixture and loss of water since there is a mass loss in TGA and DTG curves which, however, starts from 67 °C which is earlier than CaCl_2_ 2H_2_O (75 °C). The third peak starting from around 100 °C, is endothermic and broad due to the recrystallization water loss of CaCl_2_·2H_2_O.^[^ ^36^ ^]^ The mixture has a lower melting point, around 88 °C than the individual ribose (97.7 °C), evidencing the intermolecular hydrogen bond and formation of a structure that can melt. The formation of supramolecular structure could also be proven by the Powder X‐ray diffraction (PXRD) patterns of the ribose and CaCl_2_·2H_2_O mixture that shows neither characteristic peaks of ribose nor CaCl_2_·2H_2_O (Figure S1b, Supporting Information). Moreover, these supramolecular structures can reorganize to pre‐defined intermediates with adenine. As shown in Figure 1a, with the presence of CaCl_2_·2H_2_O, ribose molecules start to condense at very low temperatures (the color of the mixture changed from yellow at 5 h to extendedly‐conjugated black after 24 h), while adenine remains nonreactive. When adding ribose to the mixture of adenine and CaCl_2_·2H_2_O, the mixture became reactive. The FTIR spectrum in Figures S2 and S3, Supporting Information also reflects the reactivity of those mixtures: a number of peaks belonging to the functional group of adenine vanished after adding ribose and CaCl_2_·2H_2_O already at moderate temperatures.

### 2.2 Material Characterization

FTIR was performed to understand the chemical nature of the CNOs further. The FTIR spectra of AR6, AR7, and AR8 in Figure S4a, Supporting Information show two obvious peaks, indicating the simple constituents of the carbon frameworks. The peak at 1600 cm^−1^ is attributed to C=C/C=N groups, and the broad peak around 1250 cm^−1^ stems from the overlap of C–N and C–O groups.^[^ ^37^ ^]^ Figure S4b, Supporting Information shows the Raman spectra of AR6, AR7, and AR8. D‐band and G‐band can be seen at around 1350 and 1600 cm^−1^, which are usually associated with defective carbon structures and sp^2^‐hybridized carbon from aromatic structures, respectively. The intensity ratios of D to G peaks (*I*_D_/*I*_G_) show an almost negligible rise from 0.997 to 1.002 with the increase in pyrolysis temperature, indicating the samples keep their heteroatom‐doped, defecteous graphitic‐layered structures.^[^ ^38^, ^39^ ^]^ G′ bands are also observed, which are typical of highly ordered carbon material, demonstrating the high structural order of the graphitic system in the CNOs, while vibration disorder stems from asymmetries entered by 32 wt % of heteroatoms (Table S1, Supporting Information). TGA of these CNOs in synthetic air aims at evaluating their thermal stability (Figure S4c, Supporting Information). Apart from the mass loss at ≈100 °C that corresponds to adsorbed water, the majority of mass loss happens after 500 °C, suggesting good oxidation stability of AR6, AR7, and AR8.

**Figure 2:**
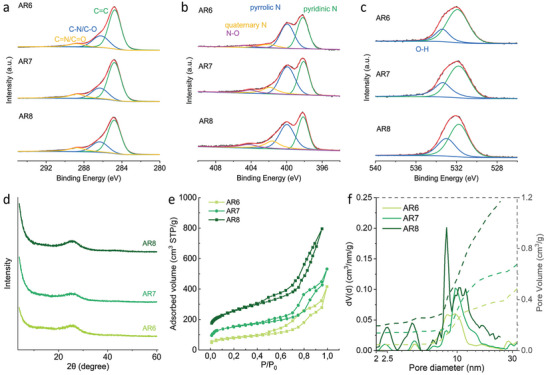
a) C 1s, b) N 1s, c) O 1s XPS spectra, d) PXRD patterns, e) N_2_ adsorption‐desorption isotherms, and f) pore distribution of AR6, AR7, and AR8.

XPS was carried out to identify the elemental compositions and their chemical state across the surface of the synthesized CNOs. The XPS survey spectra of AR6, AR7, and AR8 show the presence of the principal O 1s, N 1s, and C 1s (Figure S4d, Supporting Information). Three peaks at around 284.8, 286.3, and 288.7 eV can be fitted in the C 1s spectrum, as shown in **Figure** **2**a, which correspond to sp^2^‐hybridized carbon, C–N/C–O, and C=N/C=O, respectively. The N 1s spectrum can be deconvoluted into four peaks (Figure 2b). Peaks around 398.1, 400.0, and 401.6 eV are attributed to pyridinic‐N, pyrrolic‐N, and quaternary‐N, respectively. The peak around 404 eV is usually associated with positively charged or oxidized nitrogen functionalities.^[^ ^40^ ^]^ Yet, it can also be a collective effect of an increased HOMO level of the conjugated framework. With the increase in heating temperature, the contents of more stable quaternary‐N species and “N–O” species (i.e., electron‐poor species) are increased, as shown in Table S2, Supporting Information, while pyridinic‐N and pyrrolic‐N exhibit a downward trend. For instance, the content of quaternary‐N in AR8 is two times that of AR6, revealing the increasing electropositivity of nitrogen species in CNOs at higher pyrolysis temperatures. Though the content of different N‐species is changed, the total nitrogen content is constant, suggesting a structural conjugation change of the N‐species in the whole CNOs structure with no nitrogen loss during the pyrolysis. O 1s spectra also show the rise of O–C species from AR6 to AR8, as shown in Figure 2c, because O–H at the edge of CNOs layers tends to become a more stable C–O–C structure. Therefore, the electronic properties of the CNOs can be tailored by controlling the pyrolysis temperature.

**Figure 3:**
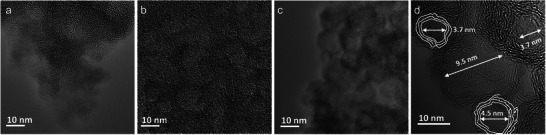
TEM images of a) AR6, b) AR7, and c) AR8. d) HRTEM image of AR8.

PXRD analyses of the obtained CNOs all show a broad peak at around 25° (Figure 2d), corresponding to the (002) planes from graphitic carbon layers stacking.^[^ ^41^ ^]^ The CNOs are partially ordered but not perfectly translationally ordered (the nanostructure is shown in TEM images below, **Figure** **3**). Thus, diffraction can only generate a broad peak instead a sharp one.

The surface area and pore size distribution of the prepared CNOs were evaluated by nitrogen adsorption/desorption isotherms. As shown in Figure 2e, AR6, AR7, and AR8 all display type IV isotherms with a distinct hysteresis loop associated with capillary condensation in mesopores. It is well acknowledged that hysteresis is attributed to thermodynamic and/or network effects. The H4 hysteresis loop represents the presence of mesopores, as also quantified with the pore distribution in Figure 2f. AR8 showed more accessible pores with the highest total pore volume, which is mainly contributed by mesopores. Similar to the mesopore volume, microporosity in the CNOs increased with increasing pyrolysis temperature. We observe a huge jump in absolute micropore volume and relative contribution: from 9.3% to 20.2% when the temperature was raised from 600 to 700 °C (**Table** **1**). Note that the micropores are the potential location of single metal atoms and are, of course, the main contribution to the specific surface area.^[^ ^42^, ^43^ ^]^

**Table 1:** Specific area, total pore volume, and percentage of different sizes of pores of the CNOs.

Sample	S_BET_ [m^2^ g^−1^]	V_micropore_ [cc g^−1^]	V_micropore_ [%]	Total pore volume [cc g^−1^]
AR6	275	0.046	9.3	0.49
AR7	545	0.138	20.2	0.683
AR8	766	0.192	21.7	0.883

Another strong evidence for the idea that CaCl2·2H2O makes ribose more active and promotes condensation is that the ratio of salt to precursors could alter the specific surface area (Table S3, Supporting Information) and composition (Table S1, Supporting Information) of produced CNOs but make no difference in the pore size distribution. As shown in Figure S5a,b, Supporting Information, CNOs showed similar pore size distribution after changing the ratio of salt to the precursor. Still, the surface area of CNOs with a higher salt/precursor ratio was larger, confirming that CaCl_2_·2H_2_O acts as a template in the synthesis process. If CaCl_2_·2H_2_O was not enough to form sufficiently reactive supramolecules with ribose and to condense with adenine, then the residual adenine molecules will condense with each other, resulting in higher nitrogen content in the produced CNOs (Table S1, Supporting Information). As shown in Figure S5c,d, Supporting Information, when the ratio of adenine and ribose is changed, the pore structure of obtained CNOs is still similar.

The morphology of the CNOs was further characterized by scanning electron microscopy (SEM) and transmission electro microscopy (TEM). As shown in Figure S6, Supporting Information, raising the pyrolysis temperature did not change the macroscale morphology. Flakes of several micrometers in horizontal size are observed in all three samples. In TEM images, a sheet‐like morphology with included mesopores is observed. As shown in Figure S7a–c, Supporting Information, AR6, AR7, and AR8 all exhibited mesoporous structures with a pore diameter below 10 nm, consistent with the pore size distribution determined from their nitrogen isotherms. As shown in Figure 3a–c, the main difference between the samples is that the pore wall structures mature with increasing temperature. The walls of AR8 are more contrasted and well organized in graphitic layers than AR6 and AR7. AR6 showed the beginning of lattice lines, coexisting with still disordered structures (Figure S7d, Supporting Information). AR7 showed the growth of the lattice lines into the nanometer region (Figure S7e,f, Supporting Information), while in AR8, the graphitic layers essentially wrapped around the former template, which is now a mesopore, as shown in Figure 3d. In the thin edges of the flakes, HRTEM can be applied, confirming that the structure is composed of onion‐ring‐like multilayer graphite rings with a layer spacing of 0.34 nm (Figure S8, Supporting Information). Moreover, the number of these graphitic layers increased with a higher amount of ribose; all samples have about the same pore morphology, while the increase of monomer is invested in thicker walls, a further chemical control parameter given into our hands by the special process.

**Figure 4:**
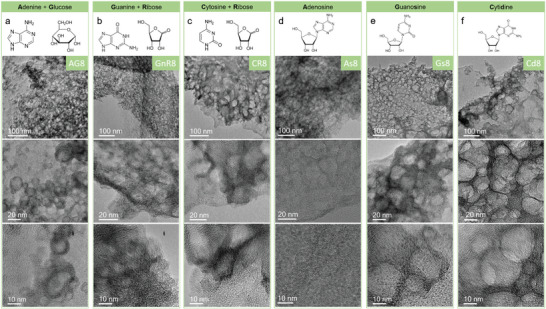
TEM images of a) AG8, b) GnR8, c) CR8, d) As8, e) Cd8, and f) Gs8.

Then we explore whether this eutectic‐mediated hard template method can be otherwise generalized, that is, if it applies to other monosaccharides and nucleobases. As shown in Figure S9a, Supporting Information, the mixture of glucose and CaCl_2_·2H_2_O (1:1 in mass) became semi‐liquid like ribose and CaCl_2_·2H_2_O after 3 h in a 90 °C oven and turned dark brown after 24 h, suggesting CaCl_2_·2H_2_O also promotes the condensation of glucose. The DSC curves of glucose showed its melting point is 165 °C, while the mixture of CaCl_2_·2H_2_O and glucose is liquid or glassy and only showed one peak assigned to water loss (Figure S9b, Supporting Information), that is, a supramolecular mixed crystal is not easily formed. In addition to the water loss, the mixed glucose and CaCl_2_·2H_2_O also showed earlier decomposition than the individual glucose and CaCl_2_·2H_2_O, as displayed in Figure S9c,d, Supporting Information, proving activation again. AG8 showed similar mesopores with graphitic‐layered structures and pore distribution with AR8, as displayed in **Figure** **4**a and Figure S10a,b, Supporting Information, indicating that our recipe can –with little variations– be extended to other sugars.

Other nucleobases, such as guanine and cytosine, were also applied to replace adenine as a nitrogen source. Again, a mesoporous morphology with wrapped graphitic walls and similar pore distribution was found (Figure 4b,c and Figure S10c,d, Supporting Information).

As we mentioned, ribose is strongly activated due to the dehydration effect of CaCl_2_·2H_2_O. While the CNOs form adenosine, ribose connected to adenine, displays a vague ring structure with some short‐range ordered structure compared to the clear graphitic rings in AR8 (Figure S11b,f,c,g, Supporting Information), indicating that the ribose in adenosine is less activated than individual ribose. But the ribose contained in adenosine can still interact with CaCl_2_ 2H_2_O and give birth to multilayer graphitic rings. Other nucleosides other than adenosine, such as guanosine and cytidine, also showed the ability to interact with CaCl_2_ 2H_2_O. The obtained CNOs from adenosine, guanosine, and cytidine –As8, Gs8, and Cd8– all exhibited the typical mesopores with graphitic margins (Figure 4d–f). Therefore, the eutectic‐mediated hard template synthesis strategy has a promising future in carbon material synthesis based on the wide possibility in the available precursors, including but not limited to aromatic compounds with hydroxyl groups. This study could also inspire further exploration of the rarely studied alkaline earth metal chlorides as templates.

The conductivity of the CNOs was measured to explore the effect of composition and structure on their electrical property. AR8, with the highest graphitic nitrogen content and ordered structure, displays the highest conductivity (AR6: 0.531 S m^−1^, AR7: 1.243 S m^−1^, AR8: 1.473 S m^−1^). This can be explained by higher quaternary‐N in AR8 because higher content of graphitic nitrogen (quaternary‐N) is favorable for the conductivity of the samples,^[^ ^44^ ^]^ together with the extended conjugation paths observed in HRTEM. And the locally ordered mesopores with graphitic rings could also be favorable for conductivity since graphitic domains, especially graphitic crystalline domains in large size, could enhance the electrical conductivity.^[^ ^45^ ^]^

Heteroatom‐doped carbons are not only active (electro‐)catalysts by themselves, due to the altered electronic structure of the carbon atoms adjacent to heteroatoms^[^ ^46^, ^47^ ^]^ but also provide a stable matrix for metal single‐atoms.^[^ ^48^ ^]^ Thus, AR8 was selected as the support to load commonly used non‐rare metals for OER electrocatalysis, nickel and iron, because of its more ordered structure, higher microporosity, and higher conductivity. A series of samples with different metal content were prepared to optimize the loading amount of metal. As expected, the Inductively coupled plasma optical emission spectroscopy (ICP‐OES) results display a positive relationship between the loading amount of Ni and the concentration of metal precursor, and the Ni content reached 16.2 wt% with a CNO/metal precursor ratio of 2 (seen in Table S4, Supporting Information). The PXRD patterns of these samples showed no characteristic peaks of Ni species, implying no severe aggregation of Ni atoms and particle formation. What is worth noting is that the Ni loading amount remains at 0.7 ± 0.03 wt% after acid etching regardless of the initial content, indicating that the highest loading capacity of CNOs for Ni at strong, non‐leachable binding sites is 0.7 wt%. Meanwhile, this deep binding capacity for Fe on CNOs is about 0.4 ± 0.02 wt%. The difference suggests a stronger interaction between AR8 and Ni than Fe. Moreover, an enhanced (electro‐)catalytic activity based on the synergy between different metals was also reported,^[^ ^49^, ^50^, ^51^ ^]^ especially Ni and Co‐based oxygen‐evolving catalysts (OECs) with Fe doping/incorporation. For instance, Cheng et al. demonstrated that bridged FeNi bimetallic twin sites significantly enhanced the OER activity.^[^ ^52^ ^]^ Thus, to analyze for a potential “Fe effect/synergy” on nickel‐based catalysts, Ni and Fe were co‐doped into AR8.^[^ ^53^ ^]^ The final contents of Ni and Fe in AR8_Ni_1_Fe_0.2__A were 0.6 and 0.04 wt%, respectively, quantifying the preferred competitive Ni‐binding.

**Figure 5:**
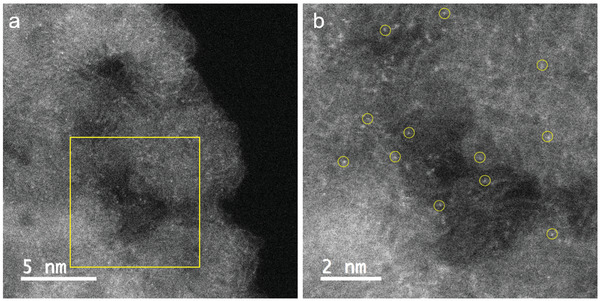
a) HAADF‐STEM image of AR8_Ni_1_Fe_0.2__A, and b) HAADF‐STEM image from (a) area.

Spherical aberration‐corrected scanning transmission electron microscopy (STEM) was used to evidence the distribution of nickel and iron nanosites. Since the signal of the high‐angle annular dark‐field (HAADF) image is approximately proportional to the square of the atomic number, and the atomic number of the metal (*Z_Ni_* = 28, *Z_Fe_* = 26) is much higher than that of C (*Z*_C_ = 6) and N (Z_N_ = 7), metallic atoms can be distinguished from the CNOs support.^[^ ^54^ ^]^ Strong signals attributable to heavier elements appear in the HAADF‐STEM image of AR8_Ni_1_Fe_0.2__A, as shown in **Figure** **5**a. Figure 5b, obtained from the yellow region in Figure 5a, displays isolated bright dots around atomic size (highlighted by yellow circles), which can confirm the subnanometer distribution of Ni and Fe in AR8_Ni_1_Fe_0.2__A. These heavy element signals can also be observed in other regions of AR8_Ni_1_Fe_0.2__A, proving that Ni and Fe are mainly distributed as nanoclusters (Figure S12, Supporting Information). The EELS signal shown in Figure S12c, Supporting Information area displays clear signals assigned to Fe and N elements and very weak signal from Ni, suggesting the existence of Fe, Ni nanoclusters.^[^ ^55^ ^]^ We exclude the potential influence from the signal of calcium as indicated by the ICPOES ‐analysis which showed a 0.008 wt% of calcium present in the sample and SEM energy‐dispersive X‐ray (EDX) spectroscopy only detected 0.015 wt% of calcium (Figure S13, Supporting Information), which is close to the limit of detection of the technique. The weak signal attributed to Ni shown in Figure S12c, Supporting Information is probably because of the low resolution of the analysis and low concentration of Ni and Fe in ca. 10 nm^2^.

### 2.3 Electrochemical Performance

**Figure 6:**
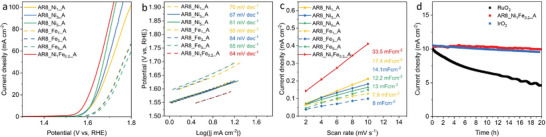
a) Linear sweep voltammetry (LSV), b) Tafel slope, and c) *C_dl_* of the catalysts. d) Current‐time (*I‐t*) chronoamperometric responses for AR8_Ni_1_Fe_0.2__A, IrO_2_, and RuO_2_.

The electrocatalytic activity of the as‐synthesized CNOs toward OER was explored by linear sweep voltammetry (LSV), as shown in Figure S14a, Supporting Information. The overpotential of the metal‐free AR6 (477 mV) is higher than the overpotential of AR7 (430 mV) and AR8 (434 mV), which can be rationalized by its low conductivity. The electrochemical stability of AR8 was assessed through a comparative analysis of its initial LSV and LSV curves after undergoing 1000 CV cycles, as illustrated in Figure S15a, Supporting Information. The absence of any activity decline indicated the exceptional durability of AR8 in electrocatalytic applications. The stability of AR8 in electrocatalysis is much higher than the benchmark RuO_2_ and comparable to commercial IrO_2_ (**Figure** **6**d). The chemical and structure stability of AR8 were further evaluated by PXRD, XPS, and TEM results of the electrode after the durability test. The PXRD pattern of AR8 demonstrated no peak shift after the durability test, indicating no expansion of the interlayer distance (Figure S16a, Supporting Information). The functionalization of AR8 was scarcely altered, as evidenced by the XPS analysis conducted after 1000 CV cycles (Figure S16b, Supporting Information). Notably, the detection of residual binder in the sample after the electrochemical assessment may have contributed to the minor discrepancies observed. Moreover, the TEM images revealed the perfect preservation of the mesoporous structure with graphitic walls of the AR8 electrode after the durability test. Therefore, given its demonstrated long‐term stability, AR8 represents a promising substrate candidate for electrocatalytic applications. Figure S14b,c, Supporting Information shows that the CNOs exhibited better OER electrocatalytic activity after loading nickel and further enhancement after adding iron. Iron doping reduced the overpotential by around 20 mV. As shown in Figure S14d, Supporting Information, the catalytic activity improved with the increasing nickel content. The overpotential of AR8_Ni_5_ is 334 mV, with nickel content reaching 16.2%, which is 90 mV lower than AR8_Ni_1,_ whose nickel content is 2.6%. This implies that the nickel species played a key role in improving the catalytic activity. Moreover, the LSV curves of AR6, AR7, and AR8 in Figure S14a, Supporting Information showed that AR6 exhibits poorer activity at higher current density regions than AR7 and AR8, which possess more porous structures. While the overpotential of AR8_Ni_1_Fe_0.2__A is 1000 mV when the current density is as high as 300 mA cm^−2^ (Figure S14e, Supporting Information), suggesting the mass transfer of the porous structure is good.

After acid etching, the overpotential of nickel‐doped CNOs is around 380 mV, which is 50 mV lower than the overpotential of pure AR8, as seen in Figure 6a (specific value is listed in Table S4, Supporting Information). The overpotentials of AR8_Ni_3_ and AR8_Ni_5_ are lower than that of AR8_Ni_3__A and AR8_Ni_5__A, suggesting the acid etching removed some active nickel species, potentially also secondary Ni‐aggregates. However, the removal of weakly bound Ni species is beneficial for the stability of the catalysts, as can be seen in Figure S15b,c, Supporting Information. The overpotential increased by 50 mV after the 1000‐cycle CV test, while AR8_Ni_5__A only showed an increase of 4 mV. The electrocatalytic activity of CNOs doped with iron was also investigated. The overpotentials of AR8_Fe_1__A, AR8_Fe_3__A, and AR8_Fe_5__A are 433, 435, and 443 mV, respectively, which shows only little improvement when compared to pristine AR8. The AR8_Ni_1_Fe_0.2__A, after co‐doping a trace amount of iron and nickel into CNOs, has an overpotential of 351 mV, a lowering by 36 and 82 mV, compared with the CNOs doped with only nickel or iron: This performance is also comparable to the benchmark RuO_2_ (358 mV in Figure S15d, Supporting Information) and other Ni and Fe single atoms‐decorated carbonaceous materials (Table S5, Supporting Information).

In addition, the kinetics of OER can be explored through the Tafel slope (Figure 6b). Tafel slopes of AR8_Ni_1__A, AR8_Ni_3__A, AR8_Ni_5__A, AR8_Fe_1__A, AR8_Fe_3__A, AR8_Fe_5__A, and AR8_Ni_1_Fe_0.2__A are 70, 67, 61, 85, 84, 84, 85, and 64 mV dec^−1^, respectively. The Tafel slopes of Ni‐Fe co‐doped CNOs and Ni‐doped CNOs are generally comparably low, with Fe‐doped species being slightly higher. The OER activity of a catalyst largely depends on the density of the accessible electrochemical sites. The density of accessible electroactive sites and the intrinsic properties of catalysts were evaluated by measuring the electrochemically active surface area. The OER catalyst acts as a capacitor when the voltage is applied: charge accumulation occurs at the electrolyte‐catalyst interface. In the absence of any Faradaic reaction, that is, before the reaction sets in, the electrode capacity is the electric double layer capacity *C_dl_*, and the electrochemically active surface area is calculated from *C_dl_*. As shown in Figure 6c, the *C_dl_* values of the iron‐doped CNOs are lower than that of Ni‐doped CNOs. This trend is consistent with the tendency of activity of these two types of catalysts, indicating that the Fe‐doped CNOs with less doping amount have a lower density of accessible catalytic active sites, while Ni‐doped CNOs have a higher density of accessible active sites and have better intrinsic activity. The *C_dl_* value of Ni‐Fe co‐doped AR8_Ni_1_Fe_0.2__A is the largest, indicating the highest active surface area.^[^ ^56^ ^]^ In practical applications, the stability of the catalyst is an extremely important parameter. The I‐t curves of AR8_Ni_1_Fe_0.2__A and RuO_2_ are compared in Figure 6d. The electrode showed a current density retention rate as high as 96.6% after 20 h at 1.6 V, which is comparable to the IrO_2_ (94.2%). In comparison, benchmark RuO_2_ showed the known decay (46.6% retention rate), indicating the much better stability of our Ni, Fe‐doped CNOs in electrocatalytic OER, outperforming in durability even well‐established materials solutions based on rare noble metals, while realizing the same performance. Besides, The TEM image of AR8_Ni_1_Fe_0.2__A after the electrocatalysis stability test showed no observed aggregation of metal clusters (Figure S17, Supporting Information).

## 3 Conclusion

In summary, adenine and ribose were used as building blocks to construct CNOs CaCl_2_·2H_2_O as a multi‐purpose reagent, then Ni and Fe‐ nanosites were deposited onto CNOs. Ribose and CaCl_2_·2H_2_O form a supermolecular eutectic solution via strong proton bridges, resulting in a homogeneous starting solution and activated dehydration and condensation of ribose molecules due to the strong acid catalysis of CaCl_2_·2H_2_O. The supermolecular approach enables to synthesize homogeneous CNOs with high oxygen and nitrogen content and stable composition in high yields. The samples are inherently mesoporous with mesopores below 10 nm due to self‐organized CaCl_2_ crystals serving as hard templates. At the same time, the walls are composed of onion‐like packed bend graphitic layers of N, O‐doped carbon. The thickness of the walls directly follows the relative ribose content.

With its inherently high specific surface area and pore volume, this sample was used as an OER electrocatalyst and already showed rather acceptable activities. Ni‐Fe co‐doping improves the OER electrocatalytic activity of CNOs in alkaline conditions, with the lowest overpotential of 351 mV and a Tafel slope of 64 mV dec^−1^. The washed catalysts showed on top a remarkable oxidation stability in long‐duration electrolysis, the real design target of the applied synthetic exercise. Now, sustainable catalysts with Ni‐Fe nanoclusters can outperform even reference materials based on rare noble metals. Additional experiments also showed that the approach is rather general and works for other carbohydrates and nucleobases.

Using supramolecular‐organized, eutectic, catalytic mixtures of ribose/carbohydrate and CaCl_2_·2H_2_O is also able to dissolve high concentrations of nucleobases as nitrogen precursors, thereby representing a simple and attractive tool for the synthesis of CNOs with stable structure, controlled meso‐and microporosity, and potentially wider compositions, all secured by chemical self‐processes.

## 4 Experimental Section

### Materials

Adenine (> 99%) and D‐(−)‐ribose (analytical grade) were purchased from TCI EUROPE N.V., and Serva, respectively. Adenosine, D‐(+)‐glucose, cytosine, cytidine, guanine, and guanosine were purchased from Aldrich. Calcium chloride‐dihydrate (≥ 99%) was purchased from Fisher Chemicals. Iron (III)‐acetylacetonate (99+%) and nickel (II)‐acetylacetonate (96%) were from Acros, respectively. Nafion (5 wt. % in a mixture of lower aliphatic alcohols and water, containing 45% water) was from Aldrich. The chemicals were all used as received. The RuO_2_ and IrO_2_ were purchased from Aldrich, 99.9%

### Synthesis of CNOs

In a typical procedure of CNOs synthesis, adenine (1.28 g) and ribose (0.78) (mole ratio = 1:1) were mixed with CaCl_2_·2H_2_O (20 g) by grinding in a mortar. The mixture was transferred to a crucible and heat‐treated at different temperatures (600, 700, and 800 °C) for 2 h with a heating rate of 1 K min^−1^ in a nitrogen atmosphere. After cooling to room temperature, the solid was washed with HCl (1 M, ≈400 mL) at room temperature 2 times and 70 °C 1 time. After washing, the material was dried at 60 °C under atmospheric pressure for several hours and then at 150 °C under vacuum overnight to remove the absorbed water. The final product was obtained with a weight yield of around 30%. The samples were named AR6, AR7, and AR8, where A represents adenine, R represents ribose, and the number means the pyrolysis temperature (e.g., 6 for 600 °C). For the CNOs obtained from other precursors, G, C, Cd, Gn, and Gs represent D‐(+)‐glucose, cytosine, cytidine, guanine, and guanosine, respectively. CNOs obtained from the different mass ratios of CaCl_2_·2H_2_O and precursors, with a number at the end representing the mass ratio. For example, AR7Ca6 means the mass ratio of CaCl_2_·2H_2_O and the precursors was 6.

### Synthesis of Ni‐Fe Decorated Catalysts

Taking AR8_Ni_1_Fe_0.2_ for example, 10 mg of nickel (II)‐acetylacetonate and 2 mg of iron (III)‐acetylacetonate were dissolved in 5 mL acetone, and then 100 mg of AR8 was added into the solution. The mixture was stirred for 4 h and was put into a 60 °C oven overnight. After the evaporation of acetone, the solid was placed in a muffle furnace, heated to 350 °C for 2 h in the air, and then named AR8_Ni_1_Fe_0.2_. After cooling to room temperature, the solid was washed with hydrochloric acid (1 M, 100 mL) for 2 days and then dried. The washed sample was named AR8_Ni_1_Fe_0.2__A, where AR8 represents the CNOs support, the number after metal represents the mass of metal precursor put in 10 mg CNOs support, and A at the end represents acid wash.

### Characterization

PXRD patterns were recorded with the Bruker D8 Advance instrument using Cu‐K*α* (*λ* = 1.5418 Å) as the radiation source. The 2*θ* ranged from 5° to 60°. X‐ray photoelectron spectroscopy was obtained on a Thermo Scientific K‐Alpha. TGA was carried out on a Thermo Microbalance TG 209 F1 Libra in a Pt crucible, using synthetic air as the gas carrier with a heating rate of 10 K min^−1^. Elemental chemical analysis was carried out using a vario MICRO cube CHNOS Elemental Analyzer (Elementar Analysensysteme GmbH, Langenselbold) in the CHNS and CHNO modes. ICP‐OES was performed on a PerkinElmer ICP‐OES Optima. 500 µL HNO_3_:HCl mixture (1:2 v%) was used to dissolve the sample (≈10 mg). SEM and EDX spectroscopy were recorded on an LEO 1550‐Gemini scanning electron microscope from Zeiss, coupled with an Oxford Instruments energy‐dispersive X‐ray detector X‐MAX. Nitrogen adsorption and desorption isotherms at 77 K and carbon dioxide isotherms at 25 K were recorded on a Quadrasorb SI apparatus. The samples were degassed at 150 °C under vacuum for 20 h before the measurements. The Brunauer–Emmett–Teller (BET) method was used to calculate the specific surface area (*S_BET_*) from nitrogen adsorption data (*P*/*P_0_* < 0.2). The total pore volume (*V_T_*) was obtained from the amount of gas adsorbed at *P*/*P_0_* = 0.995. Pore size distribution and volumes of micropores and mesopores were calculated by the quenched solid density functional theory (QSDFT) model using slit/cylindrical pore shape. TEM were obtained from FEI Talos F200X G2. HAADF and low‐angle annular dark‐field (LAADF) STEM images were acquired by an aberration‐corrected transmission electron microscope (AC‐STEM), JEOL‐2100F.

For electrode preparation, a suspension of 90 wt% of CNOs and 10 wt% polyvinylidene fluoride (PVDF) in 1 mL ethanol was sonicated for 1 h and then dried. The obtained solid was roll‐pressed into a 40 µm thick sheet and cut into a 10 mm diameter pellet for conductivity measurement, which was conducted in a four‐point probe device at room temperature. Electrochemical measurements were performed on Gamry‐potentiostat in a three‐electrode cell with 0.1 м KOH as electrolyte at room temperature. 2 mg catalyst were dispersed in 1 mL ethanol and 50 µL Nafion (5wt%), and the mixture was sonicated for 45 min. The ink was sprayed on carbon fiber paper (2 × 1 cm) to get a 1 mg cm^−2^ mass loading electrode. The as‐prepared electrode, Ag/AgCl electrode (with saturated KCl), and Pt wire were used as work, reference, and counter electrodes, respectively. Electrochemical active surface area and cyclic voltammetry from 0.95 to 1.15 V with scan rates from 2 to 10 mV s^−1^ were measured, and the current density at 1.10 V was plotted against the scan rate. The linear fitted slope was *C_dl_*.

## Conflict of Interest

The authors declare no conflict of interest.

## Supporting information

Supporting Information

## Data Availability

The data that support the findings of this study are available on request from the corresponding author. The data are not publicly available due to privacy or ethical restrictions.

## References

[1] J. S. Lee , X. Wang , H. Luo , S. Dai , Adv. Mater. 2010, 22, 1004. 20217829 10.1002/adma.200903403

[2] M. Qiao , Y. Wang , Q. Wang , G. Hu , X. Mamat , S. Zhang , S. Wang , Angew. Chem., Int. Ed. 2020, 59, 2688. 10.1002/anie.201914123 31769154

[3] J. Yin , W. Zhang , N. A. Alhebshi , N. Salah , H. N. Alshareef , Small Methods 2020, 4, 1900853.

[4] W. Wei , Z. Chen , Y. Zhang , J. Chen , L. Wan , C. Du , M. Xie , X. Guo , J. Energy Chem. 2020, 48, 277.

[5] R. Wang , J. Yang , X. Chen , Y. Zhao , W. Zhao , G. Qian , S. Li , Y. Xiao , H. Chen , Y. Ye , G. Zhou , F. Pan , Adv. Energy Mater. 2020, 10, 1903550.

[6] J. Wang , P. Zhang , L. Liu , Y. Zhang , J. Yang , Z. Zeng , S. Deng , Chem. Eng. J. 2018, 348, 57.

[7] J. Li , B. Michalkiewicz , J. Min , C. Ma , X. Chen , J. Gong , E. Mijowska , T. Tang , Chem. Eng. J. 2019, 360, 250.

[8] P. Zhang , Y. Zhong , J. Ding , J. Wang , M. Xu , Q. Deng , Z. Zeng , S. Deng , Chem. Eng. J. 2019, 355, 963.

[9] H.‐W. Liang , S. Brüller , R. Dong , J. Zhang , X. Feng , K. Müllen , Nat. Commun. 2015, 6, 7992. 26250525 10.1038/ncomms8992 PMC4918366

[10] F. K. Kessler , Y. Zheng , D. Schwarz , C. Merschjann , W. Schnick , X. Wang , M. J. Bojdys , Nat. Rev. Mater. 2017, 2, 17030.

[11] A. G. Slater , A. I. Cooper , Science 2015, 348, aaa8075. 26023142 10.1126/science.aaa8075

[12] A. Stein , Z. Wang , M. A. Fierke , Adv. Mater. 2009, 21, 265.

[13] Z. Chen , W. Yang , Y. Wu , C. Zhang , J. Luo , C. Chen , Y. Li , Nano Res. 2020, 13, 3075.

[14] S. Chen , F. Bi , K. Xiang , Y. Zhang , P. Hao , M. Li , B. Zhao , X. Guo , ACS Sustainable Chem. Eng. 2019, 7, 15278.

[15] S. Chen , T. Luo , X. Li , K. Chen , J. Fu , K. Liu , C. Cai , Q. Wang , H. Li , Y. Chen , C. Ma , L. Zhu , Y.‐R. Lu , T.‐S. Chan , M. Zhu , E. Cortés , M. Liu , J. Am. Chem. Soc. 2022, 144, 14505. 35920726 10.1021/jacs.2c01194 PMC9389578

[16] S. Chen , T. Luo , K. Chen , Y. Lin , J. Fu , K. Liu , C. Cai , Q. Wang , H. Li , X. Li , J. Hu , H. Li , M. Zhu , M. Liu , Angew. Chem., Int. Ed. 2021, 60, 16607. 10.1002/anie.202104480 33982396

[17] L. He , F. Weniger , H. Neumann , M. Beller , Angew. Chem., Int. Ed. 2016, 55, 12582. 10.1002/anie.201603198 27601266

[18] W. Xia , Catal. Sci. Technol. 2016, 6, 630.

[19] R. Arrigo , M. E. Schuster , Z. Xie , Y. Yi , G. Wowsnick , L. L. Sun , K. E. Hermann , M. Friedrich , P. Kast , M. Hävecker , A. Knop‐Gericke , ACS Catal. 2015, 5, 2740.

[20] B. Qiao , J.‐X. Liang , A. Wang , C.‐Q. Xu , J. Li , T. Zhang , J. J. Liu , Nano Res. 2015, 8, 2913.

[21] S. Chen , S. Wang , P. Hao , M. Li , Y. Zhang , J. Guo , W. Ding , M. Liu , J. Wang , X. Guo , Appl. Catal., B 2022, 304, 120996.

[22] W. Zhang , Q. Yao , G. Jiang , C. Li , Y. Fu , X. Wang , A. Yu , Z. Chen , ACS Catal. 2019, 9, 11603.

[23] J. Kossmann , D. Piankova , N. V. Tarakina , J. Heske , T. D. Kühne , J. Schmidt , M. Antonietti , N. López‐Salas , Carbon 2021, 172, 497.

[24] S. N. Talapaneni , G. Singh , I. Y. Kim , K. AlBahily , A.a.H. Al‐Muhtaseb , A. S. Karakoti , E. Tavakkoli , A. Vinu , Adv. Mater. 2020, 32, 1904635. 10.1002/adma.201904635 31608512

[25] L. Sun , H. Zhou , L. Li , Y. Yao , H. Qu , C. Zhang , S. Liu , Y. Zhou , ACS Appl. Mater. Interfaces 2017, 9, 26088. 28715170 10.1021/acsami.7b07877

[26] E. Brown , P. Yan , H. Tekik , A. Elangovan , J. Wang , D. Lin , J. Li , Mater. Des. 2019, 170, 107689.

[27] C. Ma , J. Jiang , T. Xu , H. Ji , Y. Yang , G. Yang , ChemElectroChem 2018, 5, 2387.

[28] Y. Zhang , T. Qu , K. Xiang , Y. Shen , S. Chen , M. Xie , X. Guo , J. Mater. Chem. A 2018, 6, 2353.

[29] A. P. Abbott , J. C. Barron , K. S. Ryder , D. Wilson , Chemistry 2007, 13, 6495. 17477454 10.1002/chem.200601738

[30] D. Xue , D. Zhu , H. Duan , Z. Wang , Y. Lv , W. Xiong , L. Li , M. Liu , L. Gan , Chem. Commun. 2019, 55, 11219. 10.1039/c9cc06008a 31469150

[31] X. Liu , N. Fechler , M. Antonietti , Chem. Soc. Rev. 2013, 42, 8237. 23868658 10.1039/c3cs60159e

[32] Y.a. Huang , F. Yang , Z. Xu , J. Shen , J. Colloid Interface Sci. 2011, 363, 193. 21840533 10.1016/j.jcis.2011.07.065

[33] V. da Silva Lacerda , J. B. López‐Sotelo , A. Correa‐Guimarães , S. Hernández‐Navarro , M. Sánchez‐Báscones , L. M. Navas‐Gracia , P. Martín‐Ramos , J. Martín‐Gil , J. Environ. Manage. 2015, 155, 67. 25770964 10.1016/j.jenvman.2015.03.007

[34] J. Sahira , A. Mandira , P. B. Prasad , P. R. Ram , Res. J. Chem. Sci. 2013, 3, 19.

[35] J. Pampel , A. Mehmood , M. Antonietti , T. P. Fellinger , Mater. Horiz. 2017, 4, 493.

[36] C. Barreneche , A. I. Fernández , L. F. Cabeza , R. Cuypers , Energy Procedia 2014, 48, 273.

[37] W. Xia , C. Qu , Z. Liang , B. Zhao , S. Dai , B. Qiu , Y. Jiao , Q. Zhang , X. Huang , W. Guo , D. Dang , R. Zou , D. Xia , Q. Xu , M. Liu , Nano Lett. 2017, 17, 2788. 28394621 10.1021/acs.nanolett.6b05004

[38] L. Kong , J. Zhu , W. Shuang , X.‐H. Bu , Adv. Energy Mater. 2018, 8, 1801515.

[39] L. M. Malard , M. A. Pimenta , G. Dresselhaus , M. S. Dresselhaus , Phys. Rep. 2009, 473, 51.

[40] T. Susi , T. Pichler , P. Ayala , Beilstein J. Nanotechnol. 2015, 6, 177. 25671162 10.3762/bjnano.6.17 PMC4311644

[41] C. Zhao , Q. Wang , Y. Lu , B. Li , L. Chen , Y.‐S. Hu , Sci. Bull. 2018, 63, 1125. 10.1016/j.scib.2018.07.018 36658992

[42] Z. Zhang , Y. Chen , L. Zhou , C. Chen , Z. Han , B. Zhang , Q. Wu , L. Yang , L. Du , Y. Bu , P. Wang , X. Wang , H. Yang , Z. Hu , Nat. Commun. 2019, 10, 1657. 30971769 10.1038/s41467-019-09596-x PMC6458126

[43] M. Xiao , J. Zhu , L. Ma , Z. Jin , J. Ge , X. Deng , Y. Hou , Q. He , J. Li , Q. Jia , S. Mukerjee , R. Yang , Z. Jiang , D. Su , C. Liu , W. Xing , ACS Catal. 2018, 8, 2824.

[44] E. V. Suslova , E. A. Arkhipova , A. V. Kalashnik , A. S. Ivanov , S. V. Savilov , H. Xia , V. V. Lunin , Russ. J. Phys. Chem. A 2019, 93, 1952.

[45] C. Teng , D. Xie , J. Wang , Z. Yang , G. Ren , Y. Zhu , Adv. Funct. Mater. 2017, 27, 1700240.

[46] P. Su , W. Huang , J. Zhang , U. Guharoy , Q. Du , Q. Sun , Q. Jiang , Y. Cheng , J. Yang , X. Zhang , Y. Liu , S. P. Jiang , J. Liu , Nano Res. 2021, 14, 1069.

[47] A. Zhu , P. Tan , L. Qiao , Y. Liu , Y. Ma , X. Xiong , J. Pan , Inorg. Chem. Front. 2017, 4, 1748.

[48] A. Wang , J. Li , T. Zhang , Nat. Rev. Chem. 2018, 2, 65.

[49] H. Xiao , H. Shin , W. A. Goddard III , Proc. Natl. Acad. Sci. U. S. A. 2018, 115, 5872. 29784794 10.1073/pnas.1722034115 PMC6003342

[50] N. Li , D. K. Bediako , R. G. Hadt , D. Hayes , T. J. Kempa , F. Von Cube , D. C. Bell , L. X. Chen , D. G. Nocera , Proc. Natl. Acad. Sci. U. S. A. 2017, 114, 1486. 28137835 10.1073/pnas.1620787114 PMC5321006

[51] Y. Li , C. Zhao , Chem. Mater. 2016, 28, 5659.

[52] Y. Cheng , S. He , J.‐P. Veder , R. De Marco , S.‐Z. Yang , S. Ping Jiang , ChemElectroChem 2019, 6, 3478.

[53] S. Anantharaj , S. Kundu , S. Noda , Nano Energy 2021, 80, 105514.

[54] B. Rafferty , P. D. Nellist , S. J. Pennycook , J. Electron Microsc. 2001, 50, 227. 10.1093/jmicro/50.3.227 11469411

[55] D. Deng , X. Chen , L. Yu , X. Wu , Q. Liu , Y. Liu , H. Yang , H. Tian , Y. Hu , P. Du , R. Si , J. Wang , X. Cui , H. Li , J. Xiao , T. Xu , J. Deng , F. Yang , P. N. Duchesne , P. Zhang , J. Zhou , L. Sun , J. Li , X. Pan , X. Bao , Sci. Adv. 2015, 1, 1500462. 10.1126/sciadv.1500462 PMC4672762 26665170

[56] Y. Zhang , T. Qu , F. Bi , P. Hao , M. Li , S. Chen , X. Guo , M. Xie , X. Guo , ACS Sustainable Chem. Eng. 2018, 6, 16859.

