# Supplementary material for: Oxygen‐Rich Carbon Nitrides from an Eutectic Template Strategy Stabilize Ni, Fe Nanosites for Electrocatalytic Oxygen Evolution
Source: Adv Sci (Weinh). 2023 May 28;10(22):2300526. doi: 10.1002/advs.202300526 (PMC10401138; doi:10.1002/advs.202300526)
Supplement: Supplementary file 1 — Supporting Information [file ADVS-10-2300526-s001.pdf]

ADVANCED SCIENCE
Open Access

Supporting Information

for *Adv. Sci.*, DOI 10.1002/advs.202300526

Oxygen-Rich Carbon Nitrides from an Eutectic Template Strategy Stabilize Ni, Fe Nanosites for Electrocatalytic Oxygen Evolution

Chun Li, Enrico Lepre, Min Bi, Markus Antonietti, Junwu Zhu, Yongsheng Fu and Nieves López-Salas**

Supplementary information

Oxygen-rich carbon nitrides from a eutectic template strategy stabilize Ni, Fe nanosites for electrocatalytic oxygen evolution

Chun Li[a,b], *Enrico Lepre*[b], *Min Bi*[a], *Markus Antonietti*[b], *Junwu Zhu*[a], *Yongsheng Fu**[a], *and Nieves Lopez Salas**[b]

a: Key Laboratory for Soft Chemistry and Functional Materials of Ministry of Education, Nanjing University of Science and Technology, Nanjing, 210094, China. Email: fuyongsheng@njust.edu.cn

b: Colloid Chemistry Department, Max Planck Institute of Colloids and Interfaces, Am Mühlenberg 1, 14476, Potsdam, Germany. Email: nieves.lopezsalas@mpikg.mpg.de

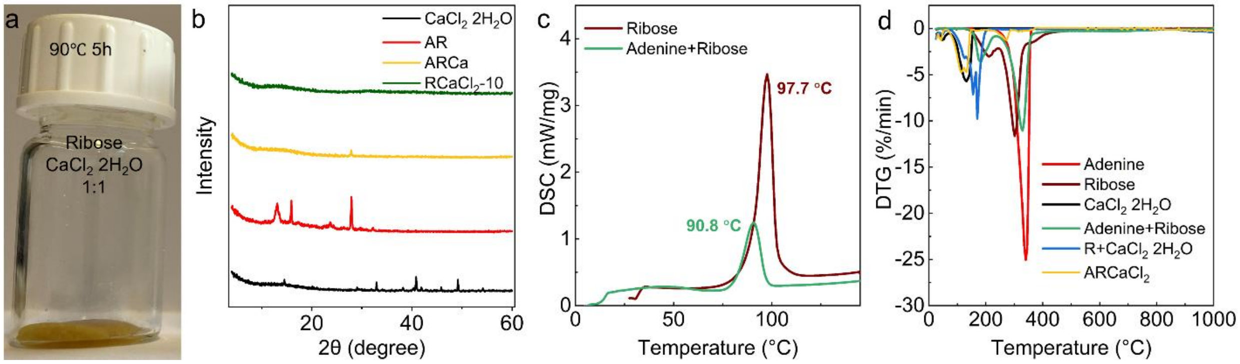

Figure S1 (a) picture of ribose and $CaCl_2$ $2H_2O$ in a 90 °C oven for 5 hours, (b) XRD, (c) DSC, and (d) DTG of the mixture of precursors and salt.

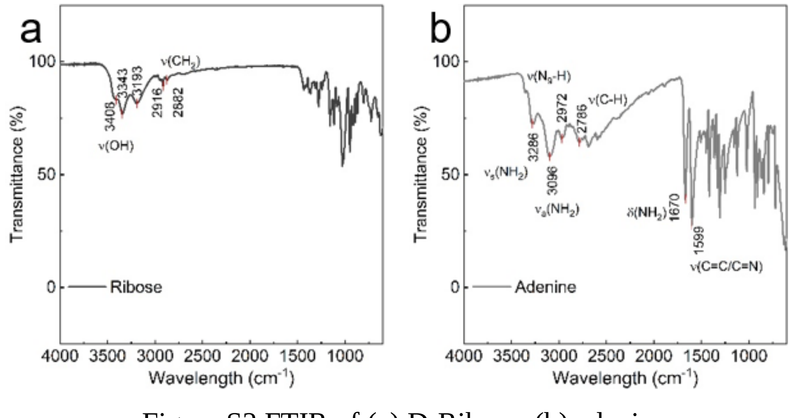

Figure S2 FTIR of (a) D-Ribose, (b) adenine

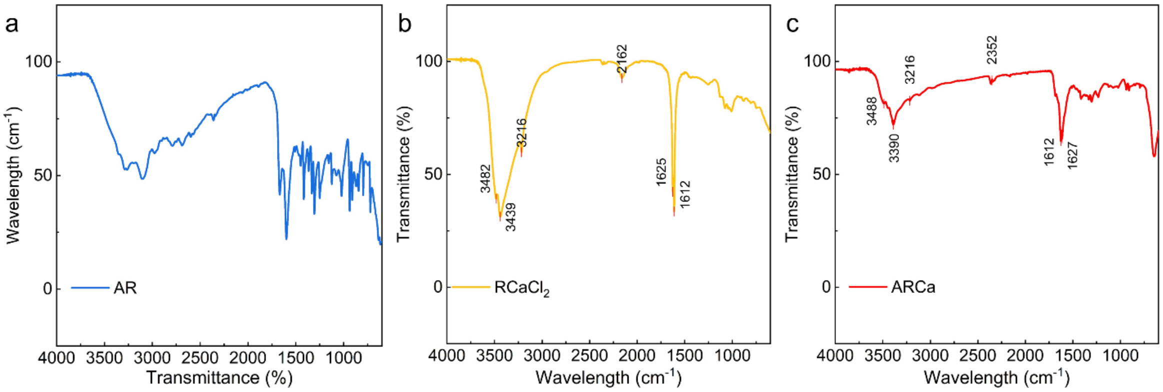

Figure S3 FTIR of (a) adenine and ribose, (b) ribose and $CaCl_2$ $2H_2O$, and (c) adenine, ribose, and $CaCl_2$ $2H_2O$.

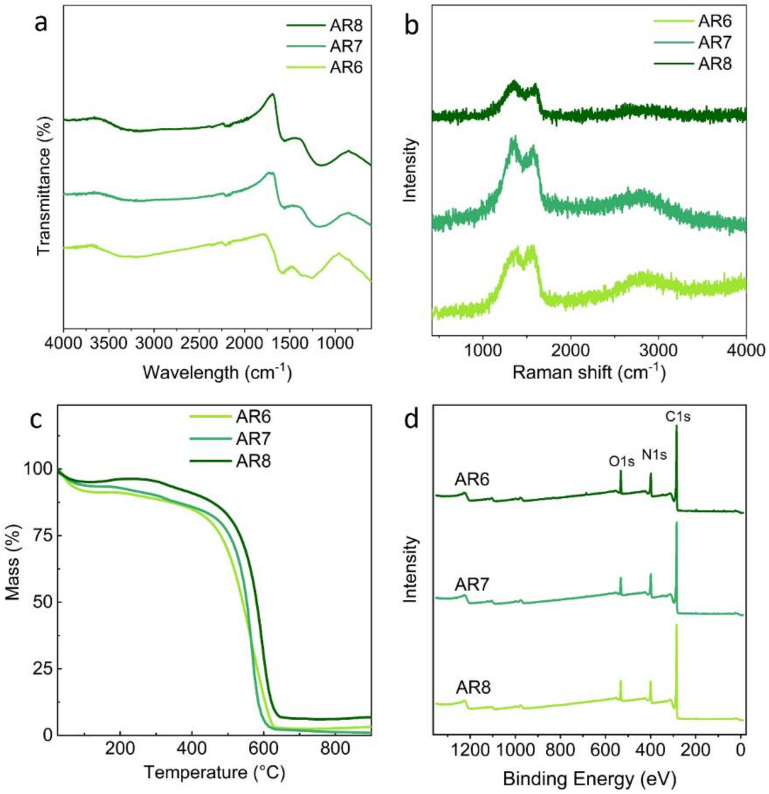

Figure S4 (a) FTIR spectra, (b) Raman spectra, (c) TGA curves, and (d) XPS survey spectra of AR6, AR7, and AR8.

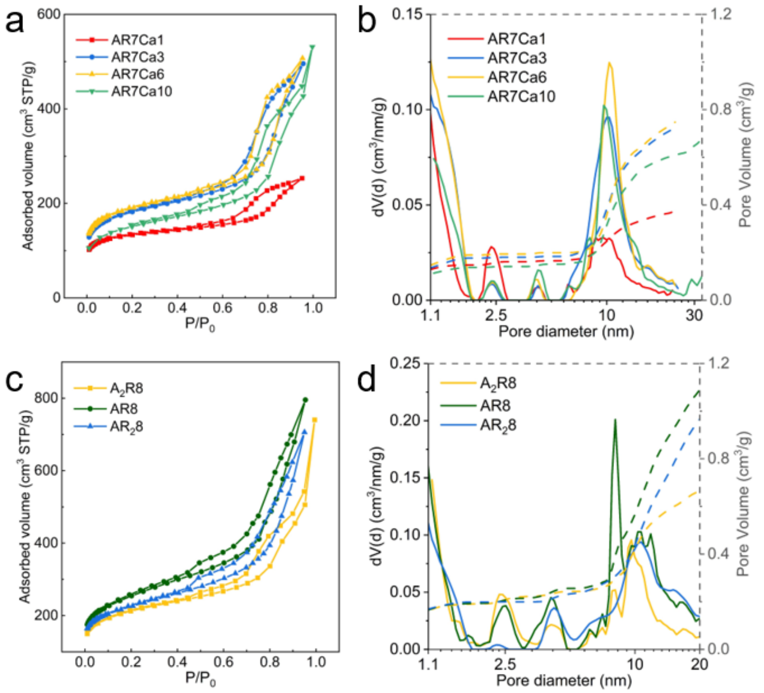

Figure S5 (a) N_2 adsorption-desorption isotherms (b) pore distribution of samples with different salt/precursor ratios and (c, d) samples with different adenine/ribose ratios.

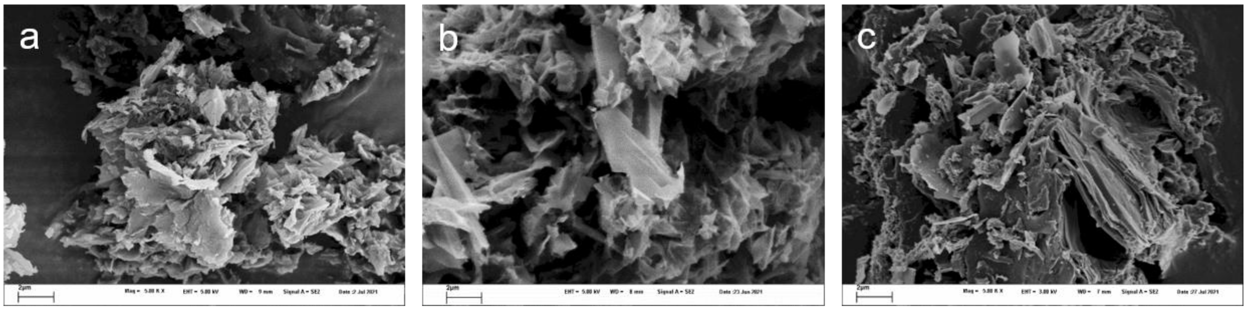

Figure S6 SEM images of (a) AR6, (b) AR7, and (c) AR8

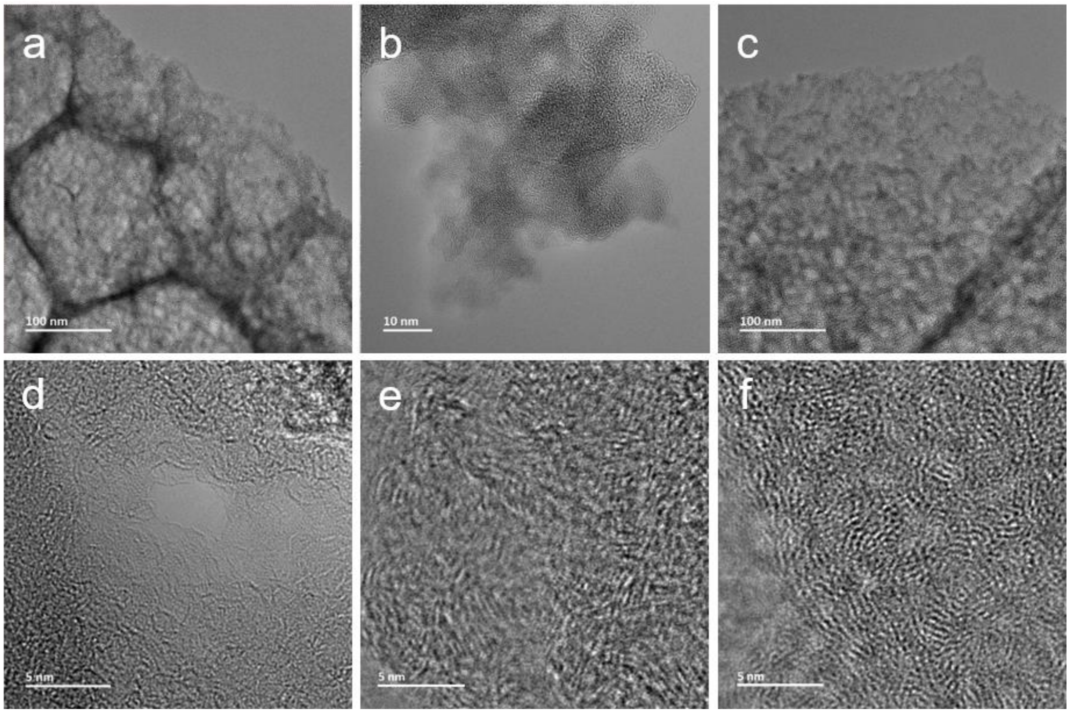

Figure S7 TEM image of (a) AR6, (b) AR7, and (c) AR8, HRTEM images of (d) AR6, and (e, f) AR7

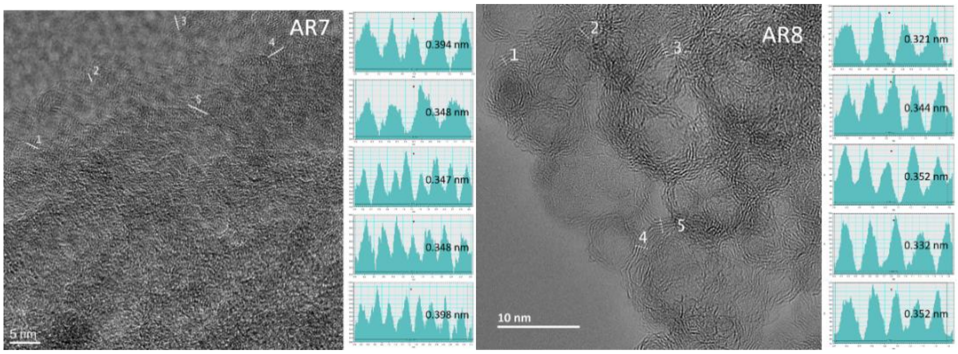

Figure S8 TEM images and high profiles of AR7 and AR8.

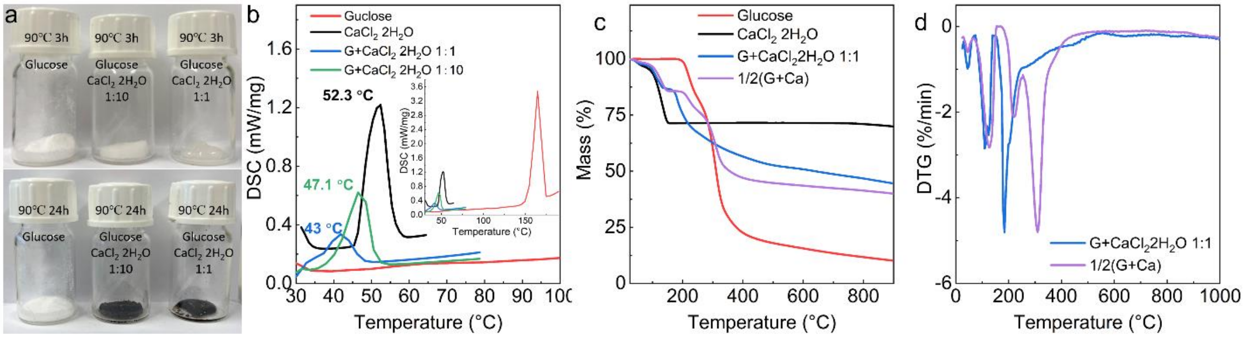

Figure S9 (a) digital photo of glucose, and the mixture with $CaCl_2 \cdot 2H_2O$ in a 90 °C oven after 3 and 24 hours, (b) DSC curves, (c) TGA curves, and (d) DTG curves of glucose, $CaCl_2 \cdot 2H_2O$ and their mixture. The label 1/2(G+ Ca) is mathematically integrated by the individual glucose and $CaCl_2 \cdot 2H_2O$.

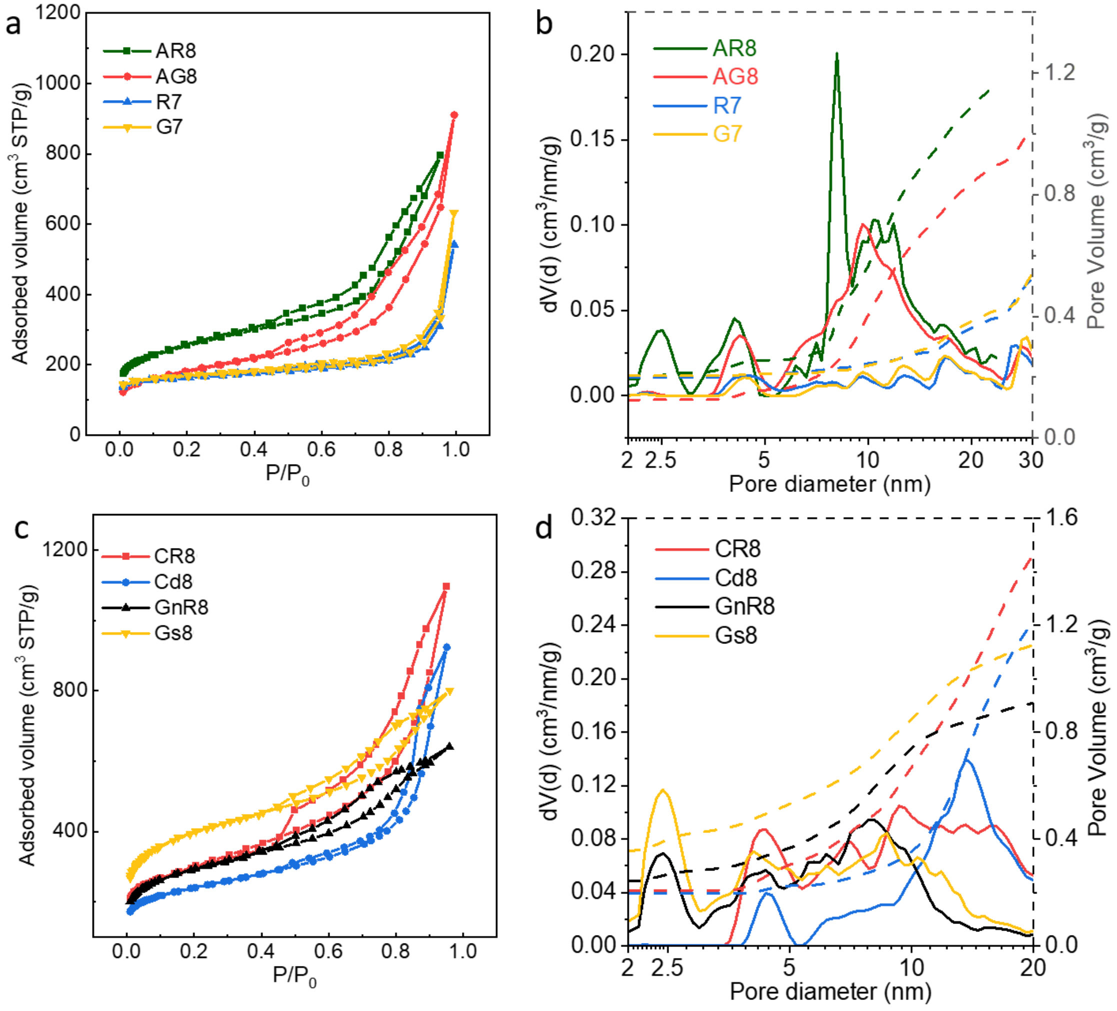

Figure S10 (a) N_2 sorption-desorption isotherms, (b) pore volume and pore distribution of AR8, AG8, R7, and G7. (c) N_2 sorption-desorption isotherms, (d) pore volume and pore distribution of CR8, Cd8, GnR8

and Gs8.

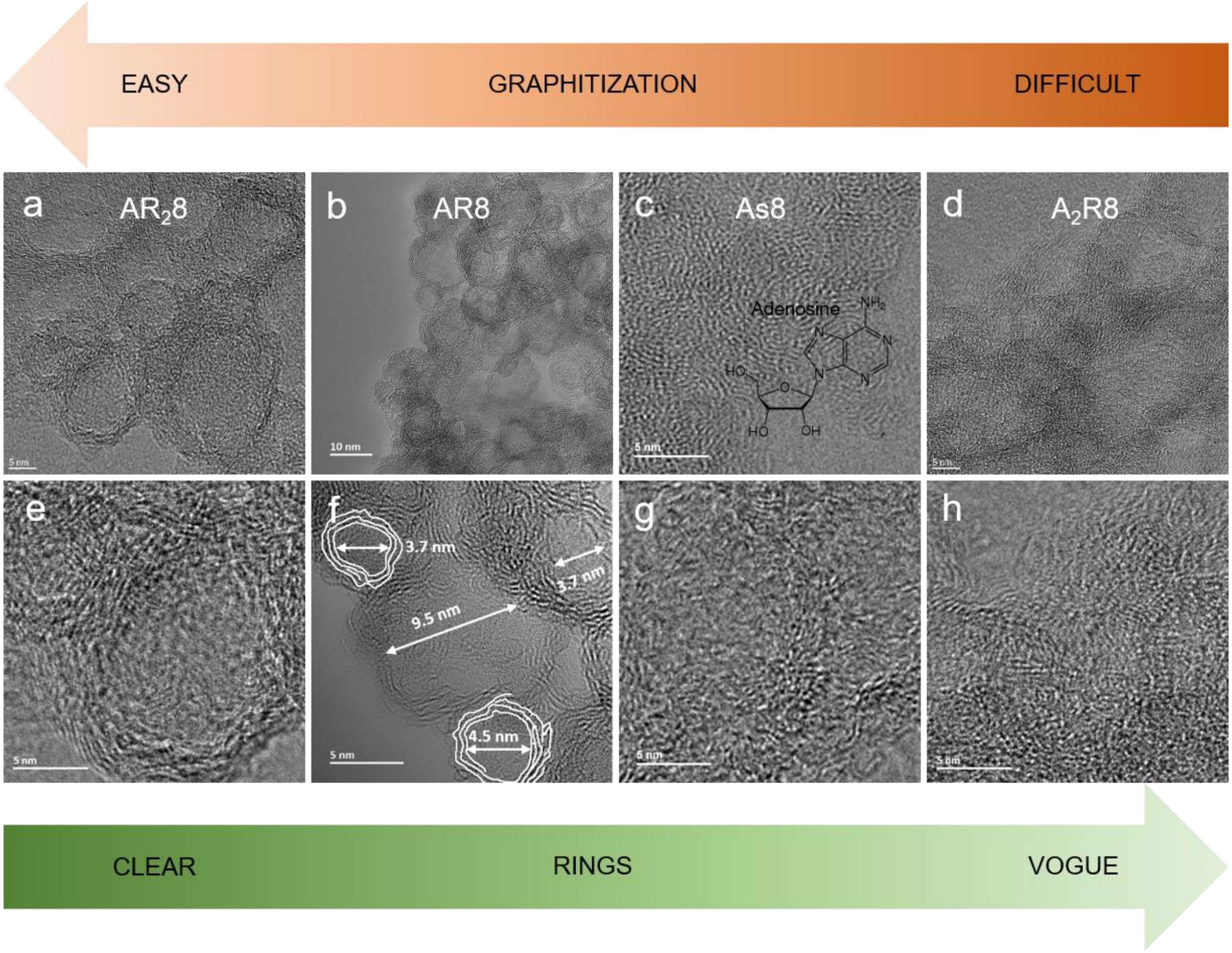

Figure S11 TEM images of (a, e) $AR_2 8$, (b, f) AR8, (c, g) As8, and (d, h) $A_2 R8$.

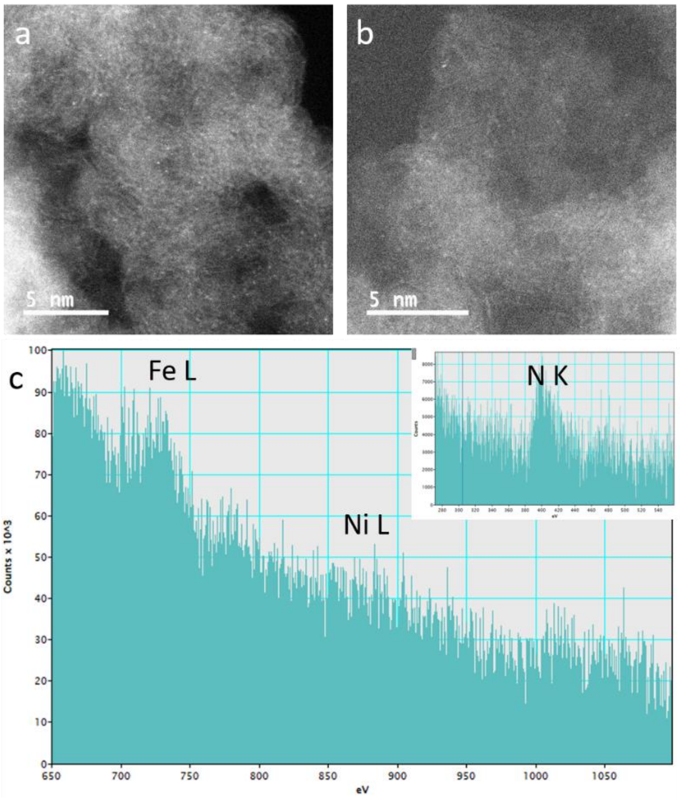

Figure S12 (a, b) HAADF-STEM images of AR8_$Ni_1Fe_{0.2}$_A. and (c) EELS atomic spectra of Fe, Ni, and N elements from the bright dots in Figure 5b.

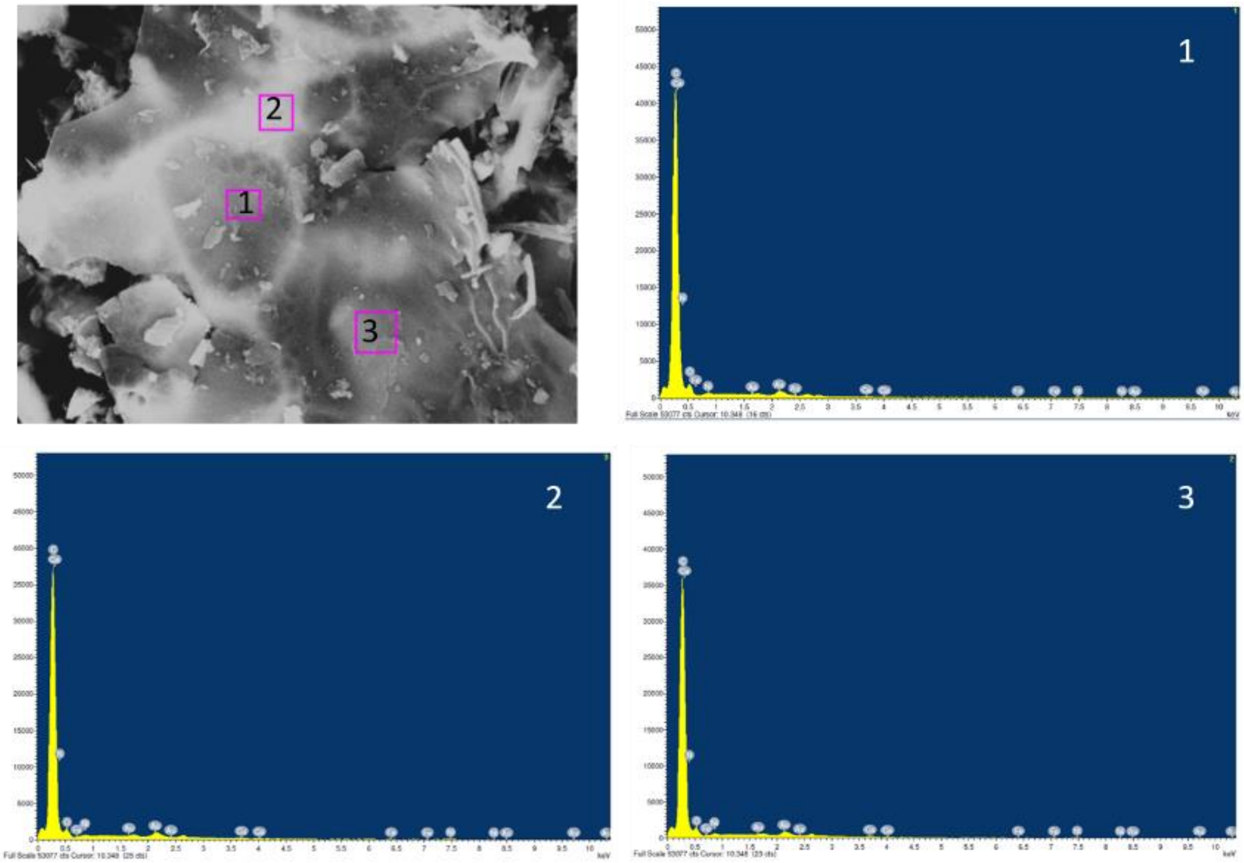

Figure S13. SEM micrograph and EDX spectrum of AR8_Ni$_1$Fe$_{0.2}$_A.

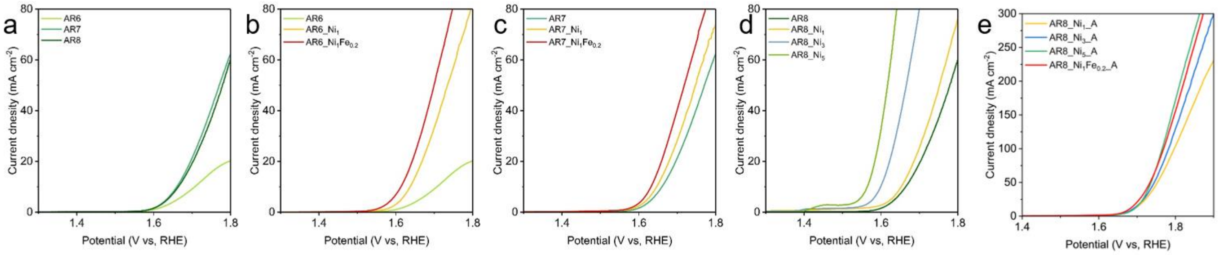

Figure S14 LSV curves of (a) CNOs, and (b-e) their comparison after loading metals.

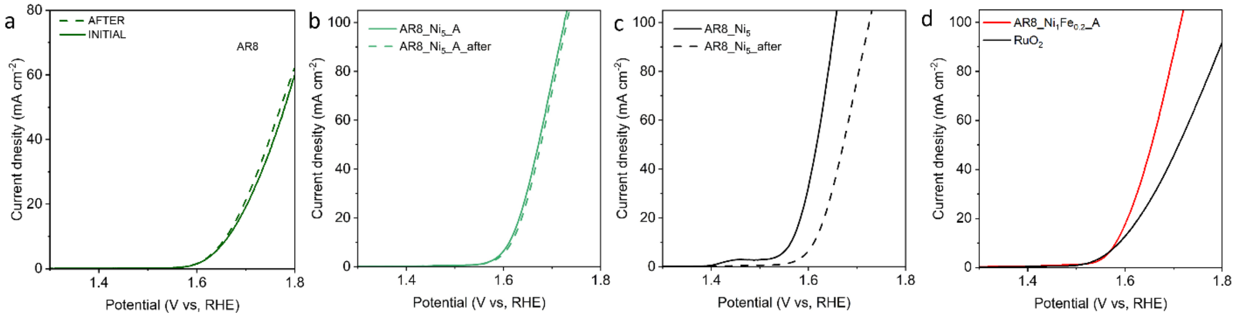

Figure S15 LSV curves of (a) AR8, (b) AR8_Ni$_5$, and (c) AR8_Ni$_5$_A before and after 1000 cycles CV and (d) comparison of AR8_Ni$_5$Fe$_{0.2}$_A and RuO_2

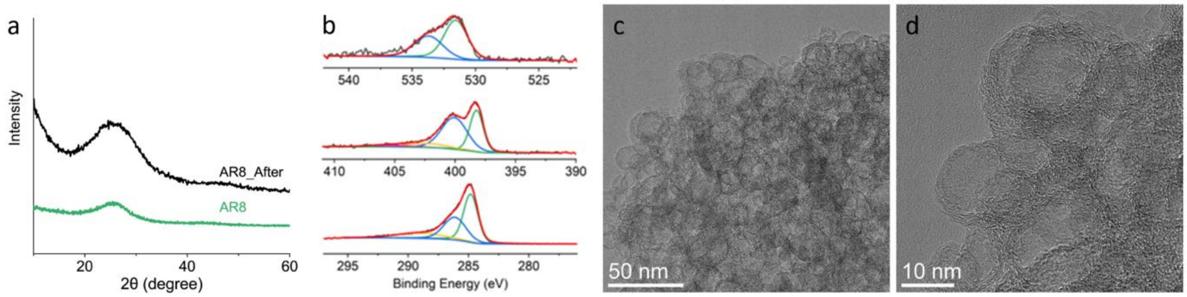

Figure S16 (a) XRD patterns of AR8 before and after durability test, (b) (Top to bottom) XPS deconvoluted O1s, N1s, C1s signals, and (c-d) TEM images of AR8 after 1000 cycles CV as durability test.

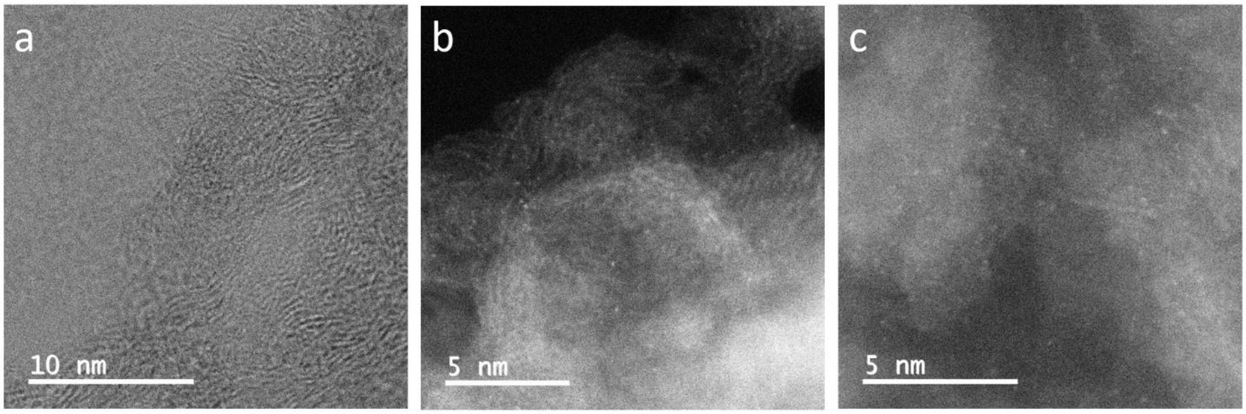

Figure S17 TEM image and HAADF-STEM images of AR8_$Ni_1Fe_{0.2}$_A after stability test.

Table S1: Element analysis results

Sample	C[wt%]	N[wt%]	O[wt%]	H[wt%]	C/N	**C/N(atomic)**	Ca
AR7Ca1	55.6	23.4	19	2	2.38	**2.78**	
AR7Ca3	59.6	25	13.4	2	2.38	**2.78**	
AR7Ca6	61.3	26	10.5	2.2	2.36	**2.75**	
AR7(Ca10)	63.8	20.5	13.1	2.6	3.1	**3.62**	0.25
AR8	63.7	20.7	13.2	2.4	3.1	**3.62**	0.27
AR6	63.8	20.5	12.75	2.95	3.1	**3.62**	0.11

Table S2 Peak information from XPS

	Sample	C1 C=C	C2 C-N	C3 C=N	N1 pyridinic	N2 pyrrolic	N3 quaternary	N4 N-O	O1 O-H	O2 O-C
Peak position	AR6	284.75	286.23	288.79	398.7	400.47	402.64	405.22	532.19	533.7
	AR7	284.78	286.32	288.74	398.3	400.1	402.09	404.3	531.99	533.54
	AR8	284.8	286.33	288.73	398.1	400	401.61	404.3	531.8	533
Atomic %	AR6	67.5	24.8	7.7	43.2	42	8.1	6.7	77	23
	AR7	65	22.2	12.8	36.1	42.1	14.3	7.5	65.7	34.3
	AR8	63.2	23.7	13.1	33.2	40.4	16.7	9.7	65.3	34.7

Table S3: Summary of surface area and pores size distribution derived from N_2 adsorption experiments.

Sample	S_{BET} [m^2/g]	Total pore volume (cc/g)
AR7Ca1	499	0.364
AR7Ca3	670	0.692
AR7Ca6	699	0.707
AR7Ca10	545	0.683
AR6	275	0.49
A_2R8	766	0.883
AR8	906	1.17
AR_28	818	1.034
AG8	641	1.01
R7	626	0.482
G7	643	0.518

Table S4 Content of metals from ICP and overpotentials of the samples

Sample	Ni[wt%]	Fe[wt%]	Ni[wt%]	Fe	Overpotential (mV) @ j=10 mA cm^{-2} Before/after etching
	Before etching		After etching		
$AR6\_Ni_1$	2.2				407
$AR6\_Ni_1Fe_{0.2}$	1.4	0.13			383
$AR7\_Ni_1$	2.7				416
$AR7\_Ni_1Fe_{0.2}$	2.8	0.18			401
$AR8\_Ni_1$	2.6		0.61		424/387
$AR8\_Ni_1Fe_{0.2}$	1.9	0.21	0.6	0.04	351
$AR8\_Ni_3$	5.7		0.73		368/383
$AR8\_Ni_5$	16.2		0.64		334/382
$AR8\_Fe_1\_A$				0.26	433
$AR8\_Fe_3\_A$				0.38	435
$AR8\_Fe_5\_A$				0.32	443

Table S5 Comparison of the OER performances of different transition-metal single atoms on carbon materials.

Catalyst	Overpotential @ j=10 mA cm^{-2}	Electrolyte	+AC	Ref
S|NiNx−PC/EG Ni: 0.2 wt.%	280	1.0 M KOH		Nat Commun 10, 1392 (2019)
Ni–NHGF (0.275 mg cm^{-2}) Ni: 0.05 at %	330	1.0 M KOH	N	Nat. Catal. 2018, 1, 63
FeCo-Nx-CN (single or multi-atom) (0.1 mg cm^{-2}) Fe: 0.40 at % ,Co: 0.12 at %	370	1.0 M KOH	N	Angew. Chem. Int. Ed. 2018, 57, 1856
S,N-Fe/N/C-CNT (0.6 mg cm^{-2}) Fe: 0.8 at %	370	0.1 M KOH	N	Angew. Chem. Int. Ed. 2017, 56, 610
A-Ni@DG (0.262 mg/cm^{-2}) Ni:1.24 wt %	270	1.0 M KOH	N	Chem 4, 285–297
NiFe@g-C_3N_4/CNT (0.382 mg/cm^{-2}) Ni: 0.84 at %, Fe:0.92 at %	326	1.0 M KOH	N	J. Mater. Chem. A, 2018,6, 6840-6846
Ni-CN-200 (79 µg cm^{-2}) Ni:2.4 at %	310@1 mA cm^{-2}	1.0 M KOH	N	Carbon 124 (2017): 180-187
Ni@NC (Ni nanoparticles) (0.4 mg cm^{-2}) Ni: 10wt %	390	0.1 M KOH	N	Adv. Energy Mater. 2015, 5, 1401660
nNiFe LDH/3D MPC (0.26 mg cm^{-2}) Ni:3.31 at%	340	0.1 M KOH	N	J. Mater. Chem. A, 2018,6, 14299-14306
NiFe LDH nanosheets	340	1.0 M KOH	N	Nano Energy, 81, 105606.
$AR8\_Ni_5$ (1 mg cm^{-2}) Ni: 0.6 wt%, Fe: 0.04 wt%	351	0.1 M KOH	**N**	**Our work**
